# Clinical experience with venetoclax in patients with newly diagnosed, relapsed, or refractory acute myeloid leukemia

**DOI:** 10.1007/s00432-022-03930-5

**Published:** 2022-01-31

**Authors:** Maximilian Fleischmann, Sebastian Scholl, Jochen J. Frietsch, Inken Hilgendorf, Karin Schrenk, Jakob Hammersen, Florian Prims, Christian Thiede, Andreas Hochhaus, Ulf Schnetzke

**Affiliations:** 1 Klinik Für Innere Medizin II, Abteilung Für Hämatologie Und Internistische Onkologie, Universitätsklinikum Jena, Am Klinikum 1, 07747 Jena, Germany; 2 Klinik Für Innere Medizin, Abteilung Für Hämatologie Und Onkologie, SRH Klinikum Burgenlandkreis Naumburg, Naumburg, Germany; 3 Medizinische Klinik I, Universitätsklinikum Carl Gustav Carus, Dresden, Germany

**Keywords:** AML, Venetoclax, Hypo-methylating agents, Refractory, Relapse, Salvage therapy

## Abstract

**Background:**

Diagnosis of acute myeloid leukemia (AML) is associated with poor outcome in elderly and unfit patients. Recently, approval of the *BCL-2* inhibitor venetoclax (VEN) in combination with hypo-methylating agents (HMA) led to a significant improvement of response rates and survival. Further, application in the relapsed or refractory (r/r) AML setting or in context of allogeneic stem cell transplantation (alloHSCT) seems feasible.

**Methods and patients:**

Fifty-six consecutive adult AML patients on VEN from January 2019 to June 2021 were analyzed retrospectively. Patients received VEN either as first-line treatment, as subsequent therapy (r/r AML excluding prior alloHSCT), or at relapse after alloHSCT. VEN was administered orally in 28-day cycles either combined with HMA or low-dose cytarabine (LDAC).

**Results:**

After a median follow-up of 11.5 (range 6.1–22.3) months, median overall survival (OS) from start of VEN treatment was 13.3 (2.2–20.5) months, 5.0 (0.8–24.3) months and 4.0 (1.5–22.1) months for first-line, subsequent line treatment and at relapse post-alloHSCT, respectively. Median OS was 11.5 (10–22.3) months from start of VEN when subsequent alloHSCT was carried out. Relapse-free survival (RFS) for the total cohort was 10.2 (2.2 – 24.3) months. Overall response rate (composite complete remission + partial remission) was 51.8% for the total cohort (61.1% for VEN first-line treatment, 52.2% for subsequent line and 42.8% at relapse post-alloHSCT). Subgroup analysis revealed a significantly reduced median OS in *FLT3-ITD* mutated AML with 3.4 (1.9–4.9) months versus 10.4 (0.8–24.3) months for non-mutated cases, (HR 4.45, 95% CI 0.89–22.13, *p* = 0.0002). Patients harboring *NPM1* or *IDH1/2* mutations lacking co-occurrence of *FLT3-ITD* showed a survival advantage over patients without those mutations (11.2 (5–24.3) months versus 5.0 (0.8–22.1) months, respectively, (HR 0.53, 95% CI 0.23 – 1.21, *p* = 0.131). Multivariate analysis revealed mutated NPM1 as a significant prognostic variable for achieving complete remission (CR) (HR 19.14, 95% CI 2.30 – 436.2, *p* < 0.05). The most common adverse events were hematological, with grade 3 and 4 neutropenia and thrombocytopenia reported in 44.6% and 14.5% of patients, respectively.

**Conclusion:**

Detailed analyses on efficacy for common clinical scenarios, such as first-line treatment, subsequent therapy (r/r AML), and application prior to and post-alloHSCT, are presented. The findings suggest VEN treatment combinations efficacious not only in first-line setting but also in r/r AML. Furthermore, VEN might play a role in a subgroup of patients with failure to conventional chemotherapy as a salvage regimen aiming for potential curative alloHSCT.

**Supplementary Information:**

The online version contains supplementary material available at 10.1007/s00432-022-03930-5.

## Introduction

Acute myeloid leukemia (AML) in elderly unfit patients remains challenging and overall long-term prognosis is poor (Juliusson et al. 2009). New treatment options especially for frontline treatment have emerged over the past years. First, introduction of hypo-methylating agents (HMAs) replaced conventional chemotherapy as first-line therapeutic option in this patient population (Dombret et al. 2015; Kantarjian et al. 2012). More recently, the B-cell leukemia/lymphoma-2 (BCL-2) inhibitor venetoclax (VEN) has been approved and led to a noteworthy impact on disease management (Apel et al. 2021; Samra et al. 2020; Pollyea et al. 2019; Kayser and Levis 2021).

Two recently published phase 3 studies demonstrated an overall survival (OS) benefit for the combination of HMA or low-dose cytarabine (LDAC) with VEN compared to single agent therapy (DiNardo et al. 2020; Wei et al. 2020).

The VIALE-A trial (NCT02993523) included 431 AML patients (286 azacitidine (AZA) plus VEN and 154 with AZA alone) with a median age of 76 years (DiNardo et al. 2020). The median OS was improved from 9.6 (7.4 – 18.7) months to 14.7 (11.9 – 18.7; HR 0.66) months with the VEN combination therapy. Attaining complete remission was more likely with AZA-VEN than with the control regime (36.7% versus 17.9%). Of note, improvement of response rates was seen across all genomic risk groups including adverse cytogenetic risk and high-risk molecular mutations.

Within another large phase 3 trial, 210 patients were treated either with LDAC alone or VEN and LDAC (Wei et al. 2020). The median OS was 4.1 months, as compared to 7.2 months within the combination arm. Although this VIALE-C trial (NCT03069352) did not meet its primary survival endpoint, the data show a potential survival benefit of the combination of VEN and LDAC and improved response rates.

Therefore, HMA-VEN combination received full U.S. Food and Drug Administration (FDA) approval in October 2020 and in May 2021 by the European Medicines Agency (EMA) as first-line therapy for patients not eligible for intensive treatment. LDAC-VEN combination has exclusively been approved by the FDA yet.

Due to improved response rates and excellent tolerability, there are ongoing debates as to whether VEN combination strategies should be implemented into first-line treatment for subsets of newly diagnosed AML patients treating in a curative intent (DiNardo et al. 2021; Maiti et al. 2021).

Compared to frontline treatment, limited data of HMA–VEN combination exist on its effect in the relapsed/refractory (r/r) setting. Encouraging results have been reported in small retrospective studies evaluating outcomes of r/r AML patients treated with VEN as single agent or in combination with other conventional agents (DiNardo et al. 2018; Aldoss et al. 2018; Piccini et al. 2021).

Here, we sought to analyze efficacy and tolerability of VEN therapy at an academic site, both in treatment-naïve and r/r AML patients. A special focus was set on common clinical settings, such as VEN application in case of induction treatment failure or at relapse following allogeneic stem cell transplantation (alloHSCT).

## Patients and methods

### Patient cohort

A total of 56 consecutive adult patients receiving VEN for AML treatment from January 2019 to June 2021 were analyzed. VEN was applied as (i) first-line therapy (*n* = 18), (ii) subsequent line (r/r AML including salvage following failure of conventional induction chemotherapy and excluding patients with prior alloHSCT) (*n* = 23), and (iii) at relapse post-alloHSCT (*n* = 15). 27 patients (48.2%) had de novo, 28 (51.8%) secondary AML (sAML) derived from myeloproliferative diseases (MPN) or myelodysplastic syndromes (MDS) and one patient (1.8%) treatment-associated AML. Treatment response was adjudicated according to European LeukemiaNet (ELN) 2017 recommendations (Döhner et al. 2017) (Table 1).

**Table 1:** Patient demographics

	*n* = 56
Sex, female (%)	24 (42.8)
Median age, years (range)	66.5 (34–83)
ECOG performance status, *n* (%)	
0–1	31 (55.4)
2–3	25 (44.6)
FAB classification, *n* (%)	
M0	0 (0)
M1/M2	15 (26.7)
M3	Excluded
M4/5	20 (35.7)
M6	2 (3.5)
M7	1 (1.8)
n.a	18 (32.1)
AML type, *n* (%)	
de novo	27 (48.2)
s-AML	28 (51.8)
t-AML	1 (1.8)
Blood count at baseline, median in 10^9^/l (range)	
WBC	12 (0–317)
RBC	4.5 (4.8 – 8)
ANC	0.39 (0 – 12.5)
Cytogenetic abnormalities, *n* (%)	
Normal karyotype	18 (32.1)
Complex aberrant	15 (26.7)
Monosomy 7	2 (3.5)
Trisomy 8	6 (10.7)
Inversion 3	3 (5.3)
5q-	2 (3.6)
Other	9 (16)
n.a	1 (1.8)
Cytogenetic risk, *n* (%)	
Favorable	0 (0)
Intermediate	34 (60.7)
Adverse	21 (37.5)
n.a	1 (1.8)
ELN risk stratification 2017, *n* (%)	
Favorable	5 (8.9)
Intermediate	17 (30.3)
Adverse	32 (57.1)
n.a	2 (3.6)
Molecular genetics at start VEN, *n* (%)	
No aberrations detected	19 (33.9)
MLL rearrangement	3 (5.3)
KMT2A-PTD	5 (8.9)
FLT3-ITD mut	8 (14.3)
FLT3-TKD mut	4 (7.1)
NPM1 mut	9 (16)
IDH1/2 mut	7 (12.5)
TP53 mut	5 (8.9)
PTPN11 mut	2 (3.6)
RUNX1 mut	5 (8.9)
Missing data	2 (3.6)

*ECOG* Eastern cooperative oncology group, *FAB* French– American–British, *s*-*AML* secondary acute myeloid leukemia, *t*-*AML* treatment-related AML, *AlloHSCT* allogeneic hematopoietic stem cell transplantation, *n.a.* not assessed, *ELN* European LeukemiaNet, *RBC* red blood count, *WBC* white blood count, *ANC* absolute neutrophil count

### Informed consent

All patients gave their written informed consent for data acquisition and analysis. All patients were included in the SAL (Study Alliance Leukemia) registry. The analysis was approved by local ethics committee of the University Hospital Jena, Germany (no. 3967–2/13 for SAL registry).

### Patients’ treatment

VEN was administered orally using a 28-day cycle either combined with HMA (decitabine 20 mg/m^2^ intravenously daily on days 1–5, 5-azacitidine 75 mg/m^2^ subcutaneously for 7 consecutive days, repeated after 28 days each) or low-dose cytarabine (LDAC 40 mg subcutaneously on days 1–7, repeated after 28 days) (Jonas and Pollyea 2019). Dose adjustment of VEN was required depending on concomitant azole therapy, tolerance and cytopenia (Jonas and Pollyea 2019).

The majority of patients undergoing alloHSCT received a reduced-toxicity conditioning (RTC) based on treosulfan or busulfan in combination with fludarabine with or without ATG prior to transplantation (*n* = 18, 81.8%) (Casper et al. 2012; Beelen et al. 2020). The remaining patients underwent myeloablative conditioning (MAC) (*n* = 4, 18.2%) (Jethava et al. 2017). Further characteristics of patients undergoing alloHSCT are summarized in Table S1.

### Safety analyses

Hematologic and non-hematologic toxicity was evaluated according to the Common Terminology Criteria and Adverse Events classification (CTCAE v5.0). For classification of hematological toxicity changes for neutrophils, platelets and hemoglobin compared to baseline values at start of VEN treatment have been assessed.

### Cytogenetic and molecular genetic analysis

Cytogenetic evaluation was performed using standard banding techniques, and karyotypes were described according to the currently valid International System for Human Cytogenetic Nomenclature (McGowan-Jordan et al. 2021). Cytogenetic categorization into favorable, intermediate or adverse risk was performed on the basis of recommended criteria (Döhner et al. 2017). Detections of AML-specific molecular aberrations according to ELN 2017 guidelines were performed by next-generation sequencing (NGS) as published previously (Stasik et al. 2020). Genetic characteristics before initiating VEN treatment have been assessed and reported in Table 1.

### Response assessment

Response assessment was carried out separately for three disease settings: first-line, subsequent line (r/r AML excluding patients who underwent prior alloHSCT) and relapse post-alloHSCT. Subgroup analyses were accomplished for patients achieving composite complete remission (CRc) and for distinct molecular aberrations (*FLT3-ITD*, *NPM1*, and *IDH1/2*).

Efficacy assessments were performed by calculation for OS, progression-free survival (PFS) and survival from start VEN treatment to last follow-up or death from any cause. OS is defined as date of first diagnosis of AML to date of last follow-up and death from any cause. PFS is defined as date of initiation of VEN until progression/relapse or last follow-up/death from any cause. Relapse-free survival (RFS) was calculated for patients attaining CRc measured from date of remission to relapse or last follow-up.

Overall response rate (ORR) was defined as CRc comprising complete remission, complete remission with incomplete hematological recovery (CRi) and complete remission with incomplete platelet recovery (CRp) and partial remission (PR). Additional response criteria were applied according to ELN 2017 guidelines (Döhner et al. 2017). CR was defined as 5% blasts or less within the bone marrow and adequate peripheral blood counts (neutrophils ≥ 1.0 × 10^9^/l, platelets ≥ 100 × 10^9^/l). Partial remission was defined as 5—25% blasts in the bone marrow and a total reduction of blasts of at least 50% of AML blasts. Progressive disease (PD) was defined as increase of bone marrow and/or peripheral blast count or new extramedullary manifestations. Stable disease (SD) was defined when CRc, PR or PD are not met and last for at least 3 months.

Additionally, transfusion requirements were also assessed for platelets and red blood cells (RBC). Transfusion dependence was determined by Gale criteria with greater than or equal to 2 units per month over the prior 3 months (Gale et al. 2011).

### Statistics

Time-to-event analyses (OS, PFS) were estimated using Kaplan–Meier method and compared using log-rank test. *p* values of < 0.05 were considered as statistically significant. Statistical analyses were performed using GraphPad Prism 8.0.2 (GraphPad Inc., San Diego, CA, USA).

## Results

### Baseline characteristics

A total of 56 adult AML patients with a median age of 66.5 (range 34–83) years were included in this analysis. Table 1 provides information about morphology, AML-type and genetics including risk stratification.

48.2% (*n* = 27) presented with de novo AML, 51.8% (*n* = 29) with secondary AML (sAML) including one patient with treatment association (Table 1). The median time of myelodysplastic syndrome (MDS) diagnosis to sAML development was 11.7 (1.7 – 156.4) months (data not shown).

Eastern Cooperative Oncology Group (ECOG) performance status rated 31 patients (55.3%) as 0–1 and 25 patients (44.6%) as 2–3, respectively.

Cytogenetic risk profiling was performed for 55 of 56 patients (98.2%) and resulted in favorable, intermediate and adverse risk in 0 (0%), 34 (60.7%) and 21 (37.5%), respectively. Data for molecular aberrations were available in 54 of 56 (96.4%) AML patients. Risk stratification according to the current ELN criteria 2017 showed a distribution in favorable, intermediate and adverse risk in 5 (8.9%), 17 (30.3%) and 32 (57.1%) of patients, respectively (Table 1). Further characteristics of those patients who received alloHSCT (39.3%, *n* = 22) are summarized in Table S1.

### Treatment characteristics

**Fig. 1:**
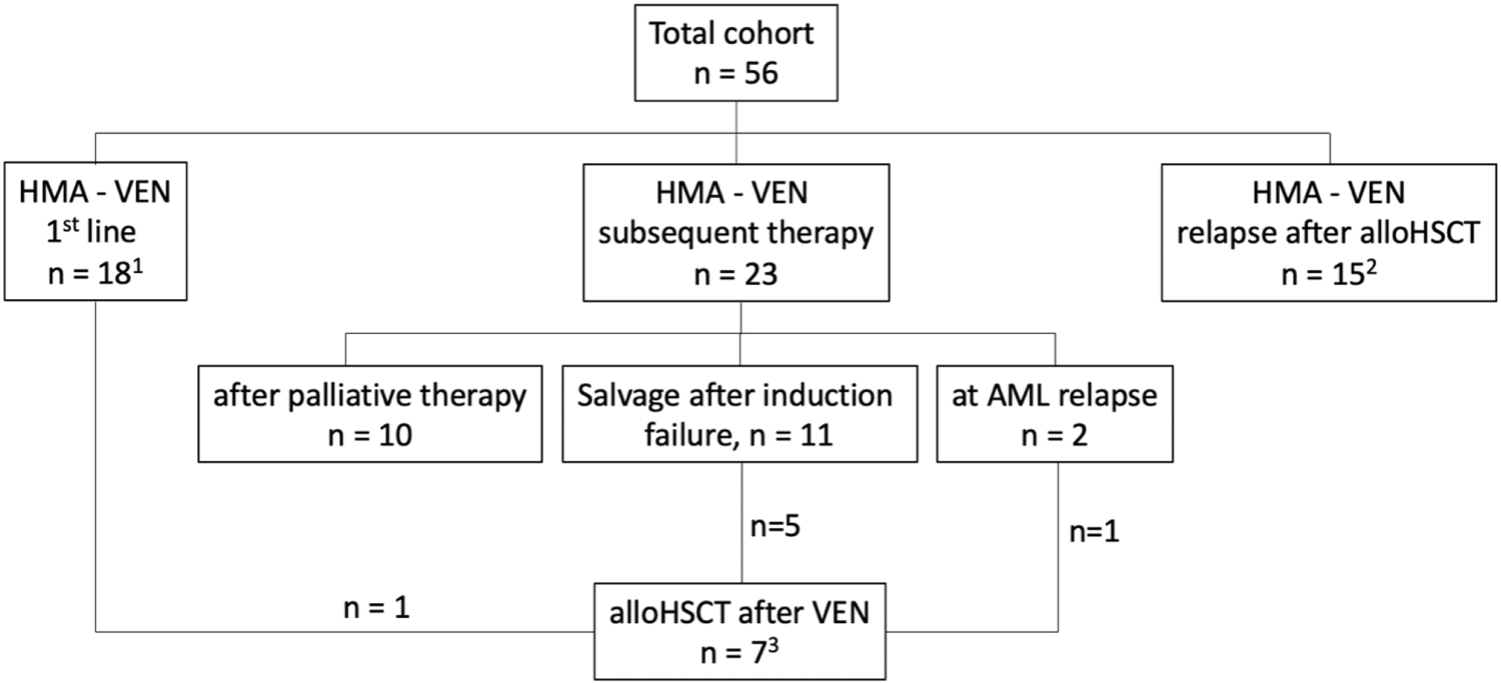
CONSORT diagram: detailed overview about different treatment cohorts and disease settings. ^1^One pt received HMA-VEN as first-line treatment with intention to alloHSCT. ^2^Including 2 pts with AML relapse after second alloHSCT. ^3^3 pts received VEN twice in relapse after alloHSCT without response (excluded from analysis). *VEN* Venetoclax, *pt(s)* patient(s), *HMA* hypo-methylating agent, *alloHSCT* allogeneic hematopoietic stem cell transplantation

Treatment intentions are illustrated in the CONSORT diagram (Fig. 1) and detailed information is provided in Table 2.

**Table 2:** Treatment characteristics

Treatment cohorts, *n* (%)	
VEN first-line, palliative	17 (30.4)
Median age	77 ( 54 – 83)
VEN > 1 line, palliative	17 (30.4)
Median age	71 (50 – 81)
VEN at relapse post-alloHSCT	15 (26.7)
Median age	56 (34 – 65)
VEN as salvage prior to alloHSCT	7 (12.5)
Median age	61 (50 – 72)
Remission status prior to VEN, *n* (%)	
Progressive disease	50 (89.2)
Partial remission	4 (7.1)
Complete remission	1 (1.8)
Stable disease	1 (1.8)
Refractory to any line prior VEN	22/38 (57.9)
Lines prior to VEN, median (range)	1.5 (0–8)
Combination with VEN, *n* (%)	
Decitabine	22 (39.3)
5-Azacitidine	28 (50)
LDAC	2 (3.6)
More than one (VEN beyond progression)*	4 (7.1)
Cycles VEN applied, median (range)	3 (1–18)
Dosing	
Months of VEN treatment, median (range)	3.4 (0.6–22.1)
Total amount applied in mg, median (range)	13,920 (640 – 106,720)
Mean dosage of VEN in mg (range)	149 (20.9 – 362,5)
Concurrent azole application, *n* (%)	48/56 (85.7)
Posaconazole	23 (47.9)
Fluconazole	11 (22.9)
Isavuconazole	9 (18.7)
More than one azole (consecutively)	5 (10.4)
VEN interruption, *n* (%)	16 (28.6)
Due to neutropenia	15 (93.7)
Due to nausea	1 (6.2)
CR achieved before VEN, median (range)	1 (0–4)
PR achieved before VEN, median (range)	0.5 (0–4)
Prior treatment lines before VEN, *n*	
HMA	22
LDAC	8
Consolidation chemotherapy	14
Induction/re-induction	44
alloHSCT	15
TKI	6
Other	9
Treatment lines post VEN, *n*	18/56
alloHSCT	7
Quizartinib	1
LDAC	7
Second VEN application	2
Clinical trial	1

*VEN* venetoclax, *alloHSCT* allogeneic stem cell transplantation, *LDAC* low-dose cytarabine, *HMA* hypo-methylating agents, *CR* complete remission, *PR* partial remission, *TKI* tyrosine kinase inhibitor

*VEN was continued beyond progression while the backbone (HMA/LDAC) was changed

For first-line therapy, VEN was applied in 18 AML patients, 23 patients received VEN as subsequent therapy (r/r AML without prior alloHSCT). A third subgroup was treated with VEN due to disease relapse following alloHSCT (n = 15). Of note, 7 patients were successfully transitioned to alloHSCT after VEN treatment (demographics and survival are reported in Table S1 and Figure S1, C, respectively). Notably, one patient received VEN/HMA as first-line treatment before subsequent alloHSCT. Since all other VEN/HMA first-line patients (n = 17) were treated within a palliative setting, the only patient underwent alloHSCT was not included in survival analysis for first-line VEN treatment group.

The majority of patients received VEN in combination with an HMA agent backbone, including 22 (39.3%) decitabine and 28 (50%) AZA (Table 2).

Median time of VEN treatment was 3.4 (0.6 – 22.1) months with a median application of 3 (1–18) treatment cycles (28 days). The overall mean drug dosage was 149 (20.9–362.5) mg. In 85.7% of all patients, concurrent azole medication was applied (posaconazole 47.9%, fluconazole 22.9% and isavuconazole 18.7%). Interruption of treatment was necessary in 16 patients (28.6%) being the majority due to grade 4 neutropenia (93.7%) and one patient due to severe nausea and emesis (6.2%).

22 of 38 patients (57.9%) were refractory to any treatment line prior to VEN. In median, one CR (range 0–4) with a median of 1.5 (range 0–8) therapy lines could be achieved prior to initiation of VEN treatment. Information on prior or subsequent treatment regimens to VEN is indicated in Table 2. Importantly, except for 3 patients receiving alloHSCT, no response to further treatment approaches after VEN could be achieved.

### Response to VEN treatment

**Fig. 2:**
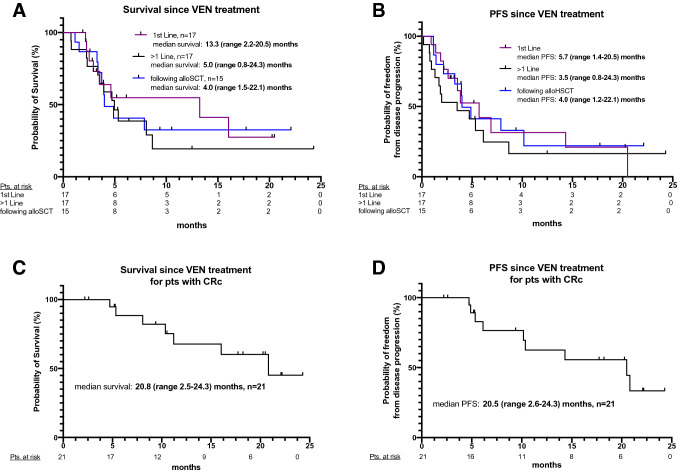
Kaplan–Meier estimates for OS and PFS were measured from starting VEN treatment in indicated groups (**A** and **B**, respectively), OS and PFS since VEN initiation in patients who achieved CRc (**C** and **D**, respectively). *OS* overall survival, *alloHSCT* allogeneic hematopoietic stem cell transplantation, *PFS* progression-free survival, *CRc* composite complete remission (CR + CRi + CRp), *VEN* Venetoclax

Subgroup analyses with respect to treatment intention showed a median OS starting at VEN treatment initiation of 13.3 (2.2–20.5) months, 5.0 (0.8–24.3) months, 4.0 (1.5–22.1) months for first-line treatment, subsequent line treatment and post-alloHSCT, respectively (Fig. 2A, B). Median follow-up was 11.5 (6.1 – 22.3) months since diagnosis of AML. Of note, survival rates for patients who received VEN prior to alloHSCT were calculated separately with a median OS of 11.5 (10.4–22.3) months (Figure S1, C).

Patients who achieved CRc on VEN treatment had a median OS of 20.8 (2.5–24.3) months and PFS of 20.5 (2.6 – 24.3) months, respectively (Fig. 2C, D).

For the whole cohort, OS starting at AML diagnosis was 18.7 (2.8 – 125.3) months and not reached in patients who received CRc (2-year OS: 63%) (Figure S1 A, B). RFS for the whole cohort was 10.2 (2.2 – 24.3) months (Figure S1, D).

**Fig. 3:**
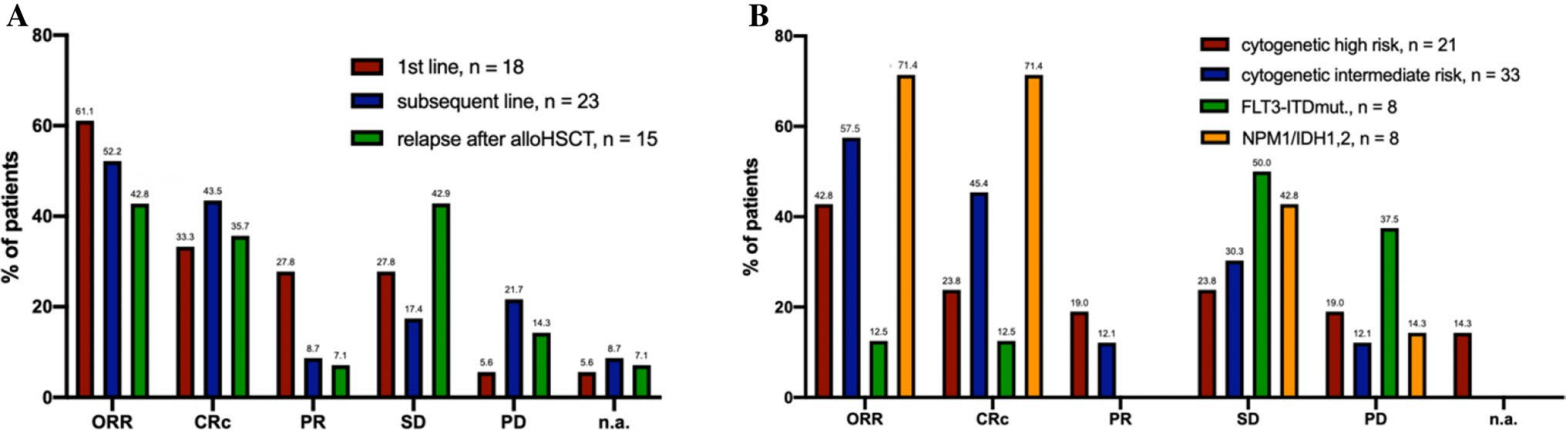
Bone marrow response rates for treatment cohorts (**A**) and distinct genetic subgroups (**B**). *alloHSCT* allogeneic hematopoietic stem cell transplantation; *ORR* overall response rate, *PD* progressive disease, *SD* stable disease, *PR* partial remission, *CRc* composite complete remission (CR + CRi + CRp), *n.a.* not assessed

An ORR of 51.8% was achieved for the whole population (data not shown). In detail, ORR of 61.1% was seen when VEN was applied as first-line treatment, 52.2% for subsequent line treatment and 42.8% at relapse post-alloHSCT (Fig. 3). Median time to first response for patients achieving CRc was 2.7 (1.4 – 16.2) months. Differences in ORR were also seen when comparing distinct genetic subgroups. Patients with cytogenetically intermediate-risk features showed an ORR of 57.5% whereas only 42.8% in high-risk patients (Fig. 3B). Presence of *FLT3-ITD* was associated with an ORR of 12.5%, in contrast 71.4% in *NPM1* and / or *IDH1,2* mutated AML, respectively (Fig. 3B). In multivariable analysis, *NPM1* mutation retained independent favorable prognostic significance with regard to achieving CRc (HR 19.14, 95% CI 2.303 – 436.2, *p* < 0.05).

**Fig. 4:**
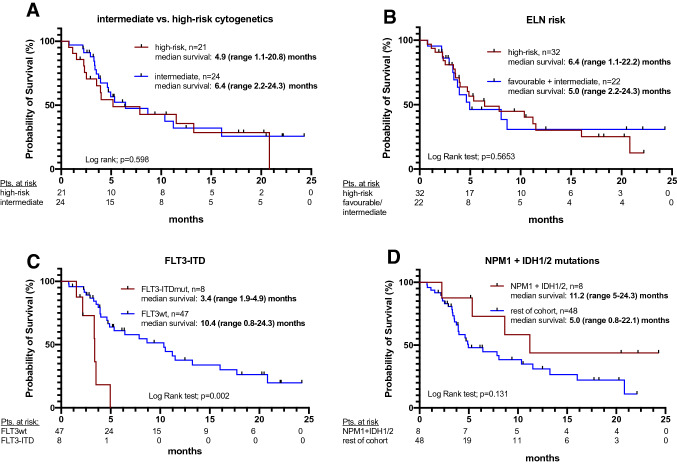
Kaplan–Meier estimates for survival time were measured from starting VEN treatment for intermediate vs. high-risk cytogenetics (**A**), ELN 2017 risk groups (**B**), according to *FLT3-ITD* (**C**) and *NPM1* or *IDH1/2* mutational status (**D**). *VEN* venetoclax, *ELN* European leukemia net

With respect to genetic subgroups, no difference in survival since VEN initiation was seen for cytogenetic (intermediate vs. high risk) and ELN risk groups (favorable + intermediate vs. high-risk) (Fig. 4A, B): 6.4 (2.2–24.3) months for intermediate vs. 4.9 (1.1–20.8) months for high-risk cytogenetics (*p* = 0.598) and 6.4 (1.1–22.2) months vs. 5.0 (2.2–24.3) months for ELN high-risk vs. favorable + intermediate, respectively (*p* = 0.565).

Analysis of distinct molecular subgroups revealed that patients harboring *FLT3-ITD* mutation (14.3%, *n* = 8) had a significantly reduced median OS of 3.4 (1.9 – 4.9) months compared to 10.4 (0.8 – 24.3) months for those without an activating *FLT3-ITD* mutation (HR 4.45, 95% CI 0.89–22.12, *p* = 0.0002).

In contrast, comparing survival of patients with *NPM1*, *IDH1*, or *IDH2* mutations without co-occurring *FLT3-ITD* mutations with the remaining cohort, an increased sensitivity to VEN-based therapy has been demonstrated: 11.2 (5 – 24.3) months versus 5.0 (0.8–22.1), respectively (HR 0.53, 95% CI 0.23 – 1.21, *p* = 0.131) (Fig. 4D).

Furthermore, assessment of blood count and transfusion (in-)dependence was performed. As demonstrated in Table 3, median count for platelets was 12 × 10^9^/l (12–317) and 42.5 × 10^9^/l (12–280) on days 0 and 100 of treatment, respectively. Transfusion dependence for platelets was decreasing from 62.9% to 47.2% and for red blood cells (RBC) from 75.9% to 55.5% during the first 100 days of treatment. Median neutrophil count was 0.39 × 10^9^/l (range 0.1–12.5) and 0.2 × 10^9^/l (range 0.1–3.8) at days 0 and 100 of treatment, respectively. On day 60, 17 of 49 patients (36%) showed an increase of the neutrophil count compared to baseline value.

**Table 3:** Development of blood count and requirement for transfusions under Venetoclax treatment

	Platelets d0 (54 pts)*	Platelets d60 (49 pts)*	Platelets d100 (36 pts)*
Median count (range) in 10^9^/l	12 (12–317)	12 (12–349)	42.5 (12–280)
Pts with requirement for transfusion	34 (62.9%)	27 (55.1%)	17 (47.2%)
Pts lost requirement for transfusion to baseline		10	+ 1

	RBC d0 (54 pts)	RBC d60 (49 pts)	RBC d100 (36 pts)
Median count (range) in 10^9^/l	4.5 (4.5–8)	4.5 (4.5–7.3)	4.5 (4.5–8.3)
Pts with requirement for transfusion	41 (75.9%)	36 (73.4%)	20 (55.5%)
Pts lost requirement for transfusion to baseline		7	+ 1

	ANC d0 (52 pts)	ANC d60 (47 pts)	ANC d100 (34 pts)
Median count (range) in 10^9^/l	0.39 (0.1–12.5)	0.2 (0.1–4.3)	0.2 (0.1–3.8)
Pts with increase of neutrophils compared to baseline		17	+ 3

*pts* patients; *d* days; *ANC* absolute neutrophils count; *RBC* red blood count

*Number of patients on treatment

### Response and survival depending on VEN dosage

The median of the mean VEN dosage of the whole cohort was 149 mg/d (20.9 – 362.5) with a median treatment duration of 105 (18 – 674) days (Table 2).

**Fig. 5:**
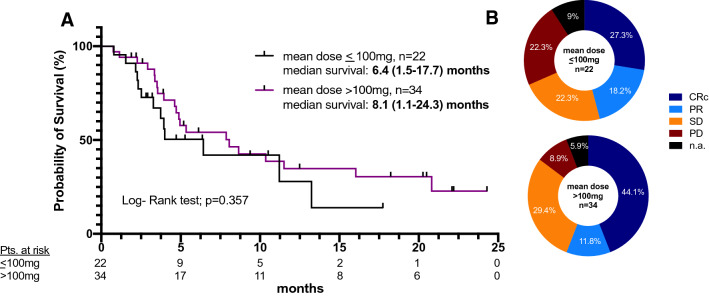
Kaplan–Meier estimates for survival time since start of VEN treatment according to mean dosage of VEN (**A**). Bone marrow response rates according to mean dosage of VEN (**B**). *CRc* composite complete remission (CR + CRi + CRp), *PR* partial remission, *SD* stable disease, *PD* progressive disease, *n.a.* not assessed, *VEN* venetoclax

To uncover a potential impact of VEN dosing on survival or response rates, patients with a mean daily dose of VEN ≤ 100 mg (*n* = 22) were compared to those with > 100 mg (n = 34). Kaplan–Meier analysis demonstrates a median survival of 6.4 (1.5–17.7) months and 8.1 (1.1–24.3) months for patients having received a mean daily dose of ≤ 100 mg and > 100 mg, respectively (p = 0.357) (Fig. 5A). Doses of > 100 mg showed an increased ORR (55.9%) compared with patients receiving ≤ 100 mg mean dosage (45.5%). Progressive disease while on VEN treatment was noted in 22.3% and 8.9% comparing the cohorts of ≤ 100 mg and > 100 mg, respectively (Fig. 5B).

### Toxicity assessment

**Fig. 6:**
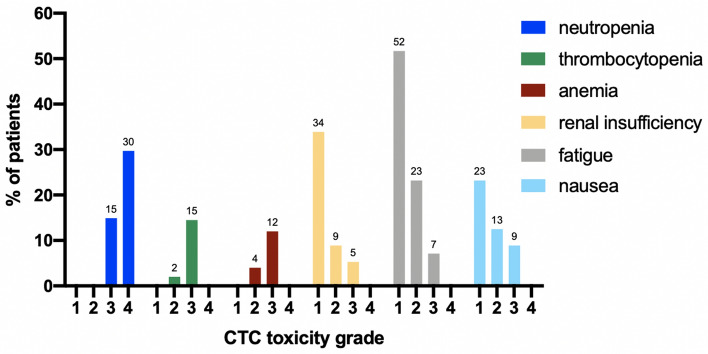
Assessment of VEN toxicities according to CTC classification. *VEN* venetoclax, *CTC* common toxicity criteria

Most frequent grade 3 and 4 side effects were hematologic with neutropenia, thrombocytopenia, and anemia (44.6%, 14.5%, 12%, respectively) (Fig. 6). Grade 3 renal insufficiency occurred in 3 patients (5.3%) including two cases of tumor lysis syndrome. One patient suffered from severe retinal bleeding, another patient from dysesthesia und body aches. Common adverse events are summarized in Fig. 6. Non-relapse mortality rate was 8.9% (5/56 patients) and early deaths till day 30 since treatment start occurred in 3.5% (2/56 patients).

## Discussion

The current study reports on 56 treatment naïve and r/r adult AML patients treated with VEN combination therapy at an academic site, outside clinical trials. Detailed analyses with respect to different disease settings were performed: VEN as first-line, subsequent line (r/r AML excluding prior alloHSCT) and VEN at relapse post-alloHSCT.

Introduction of VEN combination regimens in first-line treatment has improved survival rates in AML patients not eligible for intensive chemotherapy (DiNardo et al. 2020; Wei et al. 2020). DiNardo et al. report a median OS of 14.7 months in the first-line AZA-VEN group (286 patients) and 9.6 (7.4 – 12.7) months in the AZA control group (HR 0.66, 95% CI 0.52 – 0.85, *p* < 0.001) (DiNardo et al. 2020). In our cohort, a median OS of 13.3 (2.2 – 20.5) months for patients who were treated as first-line therapy with VEN was achieved. Furthermore, survival data are comparable to other published reports outside clinical trials. Recently, a study by Apel et al. evaluated 103 newly diagnosed AML patients and could demonstrate an OS of 9.8 (6.4 – 13.3) months (Apel et al. 2021).

As expected, differences in survival rates between subgroups were noticed. When applied as subsequent therapy (r/r AML excluding prior alloHSCT), median OS was 5.0 (0.8 – 24.3) months and in case of relapse post-alloHSCT 4.0 (1.5 – 22.1) months, respectively.

Clinical scenarios, such as failure of conventional induction chemotherapy or relapse post-alloHSCT, remain a challenge. Especially in genetically high-risk patients, re-induction by conventional chemotherapy is associated with poor response rates (Roboz et al. 2014; Mangan and Luger 2011). The use of VEN combination therapy might overcome the adverse prognosis of distinct cytogenetic and molecular aberrations. Within a small subgroup of 11 patients, VEN was applied as salvage therapy after induction failure and 5 patients were transitioned to alloHSCT. A high ORR rate of 54.5% was achieved in those 11 patients, thus exceeding ORR rates of chemotherapy-based salvage regimens in patients harboring adverse genetics (Ravandi et al. 2010). Recently, a salvage regimen combining VEN and FLAG-IDA has been demonstrated convincing response rates in r/r AML (CRc rate of 76%) providing a promising approach for this challenging patient cohort (DiNardo et al. 2021).

Another major therapeutic challenge with a high unmet need remains post-alloHSCT relapse. Relapse after alloHSCT occurs in almost half of AML patients and survival remains dismal (Schmid et al. 2018; Barrett and Battiwalla 2010). Treatment with HMAs in this setting resulted in an ORR of 19% and a low CR rate of 7%, whereas the utility of VEN in post-transplant settings is poorly studied (Motabi et al. 2016). 15 out of 56 patients of our cohort received VEN combination at relapse post-alloHSCT. Within this subgroup, 36% achieved CRc and another 7% PR. This is of importance since many of post-alloHSCT r/r AML patients are not eligible for further intensive salvage treatment. A retrospective analysis of 20 post-alloHSCT relapse patients showed a CRc rate of 70% when treated with a combination therapy consisting of VEN, LDAC and Actinomycin D (Zucenka et al. 2021). Another recently published retrospective study on r/r AML patients after alloHSCT including 29 patients demonstrated an ORR of 38% with a median OS of 2.6 months (Joshi et al. 2021). Taken together, VEN combination strategies represent a rational salvage strategy in post-alloHSCT r/r AML patients with encouraging CR rates that might offer the chance of another subsequent potential curative alloHSCT.

In palliative r/r AML patients not eligible for intensive treatment even at initial diagnosis who received VEN as subsequent line therapy, response rates and survival were considerably reduced compared to first-line therapy. Nevertheless, 43% of those r/r AML patients within our cohort attained CRc suggesting a reasonable therapeutic approach in this difficult-to-treat patient cohort. Comparable data of response rates have been reported on r/r AML patients on VEN treatment with a CR rate of 32% and a median OS of 5.5 months compared to 5.0 months in our cohort (Tenold et al. 2021). Treatment options in elderly patients at AML relapse are limited. Gilteritinib plays a role in *FLT3*-mutated AML as it is approved in the r/r setting (Perl et al. 2019). However, the frequency of *FLT3* mutations accounts for about 25% of AML patients, so a majority of patients do not benefit from the *FLT3*-inhibitor. HMAs resulted in a CR rate of 16% in r/r AML as reported in a large international patient cohort (Stahl et al. 2018). Considering the limited treatment options, our data underline the benefit of VEN-based therapy in elderly and frail r/r AML patients.

Across genetic risk groups, no difference in survival was observed when stratified by cytogenetics and ELN criteria. Detailed molecular genetic analyses revealed a significantly worse outcome in *FLT3-ITD*-mutated. Median OS from VEN to follow-up was 3.4 (1.4 – 4.9) months in *FLT3-ITD*-mutated versus 10.4 (0.8 - 24.3) months in FLT3-wildtype patients, respectively.

Conflicting data exist on the impact of FLT3-ITD-mutated AML and VEN activity. Pre-clinical models suggest that FLT3-ITD activation mediates resistance to VEN (Singh Mali et al. 2021). STAT5 activation by FLT3-ITD leads to regulation of pro-survival proteins BCL(x)L and MCL-1. These molecules are known for conferring VEN resistance; thus, combining BCL-2 inhibition and FLT3-ITD blockade might be a therapeutic rationale (Zhu et al. 2021; Maiti et al. 2021). On the other hand, DiNardo and colleagues reported a CR rate of 72% in newly diagnosed FLT3-ITD-mutated AML patients (*n* = 10) (DiNardo et al. 2020). Noteworthy, those data are based on limited patient numbers or preclinical models. Larger data sets are needed for a conclusive statement especially considering variables like allele burden of FLT3-ITD and concurrent mutations.

*NPM1*-, *IDH1*-, or *IDH2*-mutated AML (and FLT3-WT) had favorable responses to VEN which have also been documented by others (DiNardo et al. 2021). However, cautious interpretation of these molecular subgroup analyses is warranted due to the small sample size and exploratory nature.

In line with the response data, a relevant decrease in transfusion frequency could be achieved. At VEN initiation, transfusion of platelets and RBCs was necessary in 63% and 76% of patients, respectively. At day 100 of VEN treatment, 47% required platelet and 55% RBC transfusions, respectively.

When evaluating subsequent treatment strategies after VEN exposure, responses were only seen in patients undergoing alloHSCT, indicating an urgent medical need for patients progressing/relapsing on VEN treatment.

As expected, cytopenia is a major concern in VEN treatment. Particularly, 45% of patients experienced grade 4 neutropenia. Several expert opinions have been proposed to manage those commons side effects including G-CSF application, shortening of VEN treatment, reduction of VEN or HMA dose, respectively (Jonas and Pollyea 2019; Winters et al. 2019; Rausch et al. 2021). In our patient cohort treatment, interruption was performed in 29% of patients and nearly exclusively due to neutropenia. Importantly, co-administration of CYP3A4 inhibiting azoles should be addressed with a dose reduction of VEN since CYP3A4 is the primary enzyme responsible for the metabolism of VEN (Rausch et al. 2021).

By comparing probability of survival in patients who received a mean dose of 100 mg or less (22 patients) compared to doses higher than 100 mg (34 patients), no significant difference was seen with a median survival since VEN treatment of 6.4 (1.5–17.7) months or 8.1 (1.1–24.3) months, respectively. Although not statistically significant, patients treated with higher doses of VEN had a greater likelihood of achieving a CRc (44% versus 27%). Measuring individual VEN plasma concentrations and considering concurrent azole medication would yield a much more reliable result for exposure–efficacy relationship. Furthermore, genetic heterogeneity of AML, such as upregulation of *BCL2A1* and *CLEC7A* or mutations of *PTPN11* and *KRAS*, confers resistance to VEN, which has not been considered (Zhang et al. 2020).

In conclusion, VEN treatment shows impressive response rates when applied as first-line treatment suggesting VEN-based combination approaches also for curative intended AML patients either characterized by relevant comorbidities or harboring unfavorable cytogenetic risk factors associated with a high rate of induction failure. Furthermore, in r/r AML including relapse after alloHSCT, it represents a reasonable therapeutic approach. Those who fail on VEN treatment and are not eligible for intensive therapy have a poor prognosis and alternative treatment strategies are required.

## Supplementary Information

Below is the link to the electronic supplementary material.

Supplementary file1 (DOCX 91 KB)

## Data Availability

Patient records of University Hospital Jena.
