# Supplementary material for: Clinical experience with venetoclax in patients with newly diagnosed, relapsed, or refractory acute myeloid leukemia
Source: J Cancer Res Clin Oncol. 2022 Jan 31;148(11):3191–202. doi: 10.1007/s00432-022-03930-5 (PMC9508061; doi:10.1007/s00432-022-03930-5)
Supplement: Supplementary file 1 — Supplementary file1 (DOCX 91 KB) [file 432_2022_3930_MOESM1_ESM.docx]

**Supplement**

**Table S1:** Characteristics of VEN patients undergoing alloHSCT (prior to or following VEN treatment)

| Characteristics alloHSCT, n= 22/56 | n (%) |
| --- | --- |
| VEN prior to alloHSCT | 7 (12.5) |
| Sex, female | 3 (42.8) |
| Age (range) | 61 (50 – 72) |
| ELN high risk | 7 (100) |
| sAML | 4 (57.1) |
| Disease status prior to alloHSCT |  |
| CR1 | 5 (71.4) |
| PD | 2 (28.6) |
| VEN at relapse following alloHSCT | 15 (26.8) |
| Disease status prior to alloHSCT |  |
| CR1 | 13 (59.1) |
| CR2 | 3 (13.6) |
| CR3 | 1 (4.5) |
| PR | 2 (9.1) |
| PD | 2 (9.1) |
| SD | 1 (4.5) |
|  |  |
| CR after alloHSCT (at day +30) | 20 (91) |
| PD after alloHSCT (at day +30) | 2 (9) |
|  |  |
| Conditioning regimen |  |
| RTC | 18 (81.8) |
| *Treosulfan/Fludarabin/ATG* | 12 (66.6) |
| *Treosulfan/Busulfan/ATG* | 1 (5.5) |
| *FLAMSA* | 1 (5.5) |
| *Treosulfan/Fludarabin/ATG/Cytarabine* | 4 (22.2) |
| MAC | 4 (18.2) |

**Abbreviations:** *ELN* European leukemia net; *sAML* secondary acute myeloid leukemia, *CR* complete remission; *PR* partial remission; *PD* progressive disease; *SD* stable disease, *alloHSCT* allogeneic hematopoietic stem cell transplantation; *RTC* reduced toxicity conditioning; *ATG* anti-thymocyte globulin; *MAC* myeloablative conditioning; *NMAC* non-myeloablative conditioning

**Figure S1**

***Figure S1:***

*OS illustrated since initial diagnosis for whole population and for CRc patients (A and B respectively); survival since start of VEN treatment for pts who received VEN as salvage regimen prior to alloHSCT (C); RFS for the total cohort (D).*

***Abbreviations:*** *OS overall survival; VEN Venetoclax; CRc composite complete remission (CR+CRi+CRp); alloHSCT allogeneic hematopoietic stem cell transplantation; RFS relapse-free survival*

**Figure S2**

**Figure S2**

Multivariate analysis for achievement of composite complete remission (CRc)

**Abbreviations**: *ELN* European leukemia net, *sAML* secondary acute myeloid leukemia
